# 2-(2-Hydroxy-5-nitrobenzylidene)-1,3-indanedione *versus* Fluorescein Isothiocyanate in Interaction with Anti-hFABP Immunoglobulin G1: Fluorescence Quenching, Secondary Structure Alteration and Binding Sites Localization

**DOI:** 10.3390/ijms14023011

**Published:** 2013-01-31

**Authors:** Dana Stan, Carmen-Marinela Mihailescu, Mihaela Savin, Iulia Matei

**Affiliations:** 1 National Institute for R&D in Microtechnologies (IMT), 126A Erou Iancu Nicolae, Bucharest 077190, Romania; 2 DDS Diagnostic, Gh. Sincai Street No. 9A, Bucharest 040312, Romania; E-Mail: carmen_mihail28@yahoo.com; 3 Department of Physical Chemistry, Faculty of Chemistry, University of Bucharest, Bd. Regina Elisabeta, No. 4–12, Bucharest 030018, Romania; 4 Telemedica, Str. Ion Calin, No.13, Bucharest 020533, Romania; E-Mail: mihaelasvn@yahoo.com

**Keywords:** indane-1, 3-dione derivative, fluorescein isothiocyanate, immunoglobulin G1, fluorescence spectroscopy, circular dichroism, molecular modeling

## Abstract

The first step in determining whether a fluorescent dye can be used for antibody labeling consists in collecting data on its physical interaction with the latter. In the present study, the interaction between the 2-(2-hydroxy-5-nitrobenzylidene)-1,3-indanedione (HNBID) dye and the IgG1 monoclonal mouse antibody anti-human heart fatty acid binding protein (anti-hFABP) has been investigated by fluorescence and circular dichroism spectroscopies and complementary structural results were obtained by molecular modeling. We have determined the parameters characterizing this interaction, namely the quenching and binding constants, classes of binding sites, and excited state lifetimes, and we have predicted the localization of HNBID within the Fc region of anti-hFABP. The key glycosidic and amino acid residues in anti-hFABP interacting with HNBID have also been identified. A similar systematic study was undertaken for the well-known fluorescein isothiocyanate fluorophore, for comparison purposes. Our results recommend HNBID as a valuable alternative to fluorescein isothiocyanate for use as a fluorescent probe for IgG1 antibodies.

## 1. Introduction

Recent studies have demonstrated that heart-type fatty acid binding protein (hFABP) can be used as an early marker for diagnosis of acute myocardial infarction and prognosis [1,2], and other myocardial events [3]. In recent years, the efforts to improve the performance of immunoassays for fluorescence microarray of cardiac markers detection have gained considerable momentum [4]. Antibodies, labeled by chemical conjugation with fluorescent dyes, bind to the antigen of interest, allowing antigen detection by means of fluorescence techniques.

One of the most widely used classes of fluorescent labeling agents consists of fluorescein and its derivatives, including fluorescein isothiocyanate (FITC) and tetramethylrhodamine isothiocyanate. Most labeling strategies use the reaction of FITC with one of three target nucleophiles in the antibody, namely amines, sulfhydryls groups, and phenolate ions of tyrosine residues. The FITC compound is almost entirely selective for covalent modifying of ɛ- and *N*-terminal amines in proteins [5]. The main drawback to this labeling strategy is that, usually, it results in a significant decrease of the antigen-binding activity of the labeled antibody because the amino groups distributed in the Fab region can react with FITC [6]. This covalent interaction, combined with FITC physical adsorption in this region, may significantly affect the availability of the binding sites on the antibody. Many other fluorescent molecules have been synthesized and described in the literature, however only a handful came to being used as labeling agents due to drawbacks such as low fluorescence quantum yield and insufficient photostability.

During the last few years, a series of indanedione compounds were synthesized [7] and reported in the literature for their fluorescent properties [8,9]. Although substituted indane-1,3-dione derivatives have been used in the fields of medicine [10] and biology [11], literature data on their chemical and physical interactions with proteins is scarce [12,13], and no chemical and physical studies of their interaction with immunoglobulins, and their applicability as fluorescent labels were found. Moreover, very few studies deal with the theoretical characterization of the small ligand–immunoglobulin interaction [14,15].

**Scheme 1:**
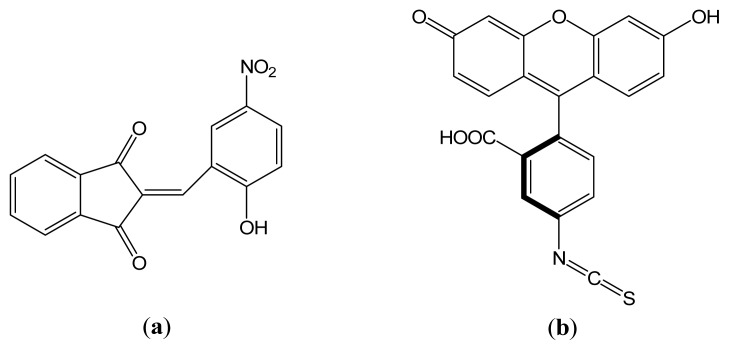
Molecular structures of (**a**) 2-(2-hydroxy-5-nitrobenzylidene)-1,3-indanedione (HNBID) and (**b**) fluorescein isothiocyanate (FITC).

In the present study we investigated the interaction of the fluorescent dye, 2-(2-hydroxy-5-nitrobenzylidene)-1,3-indanedione (HNBID, Scheme 1), with the immunoglobulin G1 (IgG1) isotype of monoclonal mouse anti-hFABP antibody of the 9F3 family (hereafter referred to as anti-hFABP), by combining two experimental spectroscopic methods (fluorescence and circular dichroism) with molecular modeling computations. A similar study was also undertaken employing FITC as ligand, for comparison purposes. Our results indicate HNBID to be a valuable alternative to the use of FITC for anti-hFABP labeling, and its further applicability in fluorescent microarray detection of hFABP protein cardiac marker will have to be investigated.

## 2. Results and Discussion

### 2.1. Effects of HNBID and FITC upon the Fluorescence Emission of Anti-hFABP

#### 2.1.1. Steady-State Fluorescence

**Figure 1:**
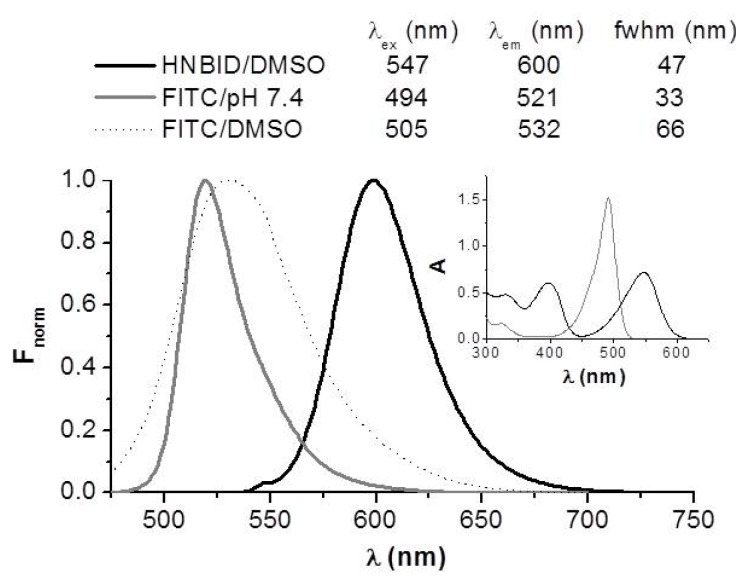
Fluorescence (normalized) and absorption (inset) spectra of HNBID and FITC in organic and aqueous environments. [HNBID] = [FITC] = 30 μM. fwhm = full width at half maximum of the band height.

Before performing a comparative study of the effects of HNBID and FITC on the fluorescence characteristics of anti-hFABP, the photophysics of the isolated ligands has been investigated in dimethyl sulfoxide (DMSO) and phosphate buffer of pH 7.4, the media most frequently used *in vitro* to mimic *in vivo* systems [16], and in anti-hFABP solution. It was observed that HNBID is highly fluorescent in DMSO (Figure 1) and practically non-fluorescent in phosphate buffer. Reversely, FITC fluoresces strongly in aqueous solution but presents weaker emission and a broader bathochromically-shifted band in the organic solvent. These results bring about the possibility of using HNBID as a valuable alternative to FITC for investigating processes in which DMSO is the medium of choice. An advantage of HNBID is that it covers a spectral range distinct from that of FITC, and thus can be employed in cases when band superposition renders FITC unusable. Moreover, in our measurement range ([HNBID]/[anti-hFABP] = 0–13), the characteristics of the fluorescence emission of HNBID were not altered by the presence of anti-hFABP.

**Figure 2:**
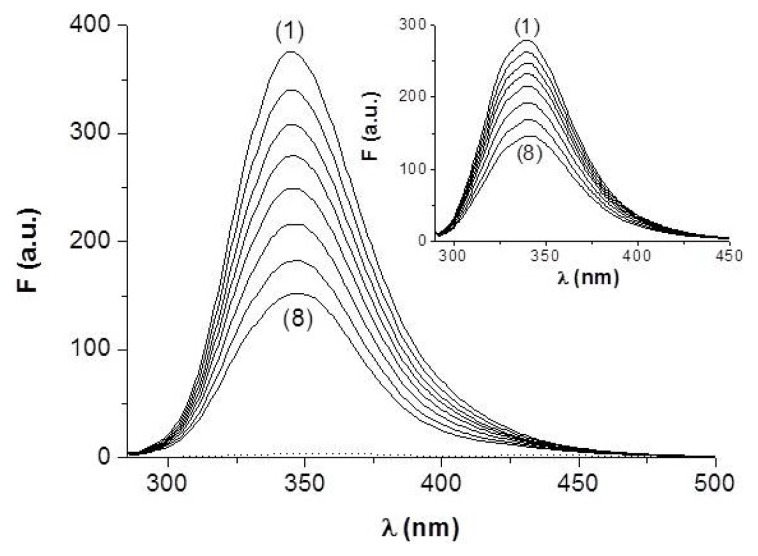
Fluorescence spectra of anti-hFABP (0.85 μM) in absence (1) and presence (2–8) of increasing concentrations of HNBID or FITC (inset) in the range 0–11 μM. Dotted spectrum: [HNBID] = 30 μM, λ_ex_ = 280 nm.

In the following, we aimed to investigate the effects of the two ligands upon the emission characteristics of anti-hFABP. The protein fluorescence band is located at 345 nm and arises from contributions of the aromatic amino acids, mainly tryptophan, Trp (λ_em_ = 340 nm), and tyrosine, Tyr (λ_em_ = 315 nm) [17]. In presence of either HNBID or FITC, the anti-hFABP fluorescence intensity decreases (Figure 2) and, on this basis, one can estimate the characteristic parameters of the interaction: the Stern-Volmer quenching constant, indicating which of the two fluorophores is the most efficient quencher, and the binding parameters (number of classes of binding sites and binding constants), indicative of the stability of the respective associations.

**Figure 3:**
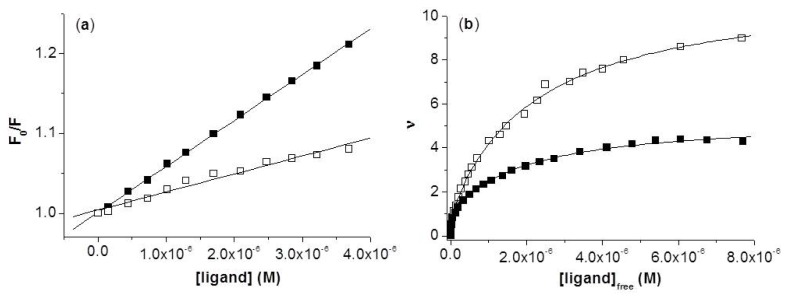
Determination of the quenching (**a**) and binding (**b**) parameters of the HNBID–anti-hFABP (■) and FITC–anti-hFABP (□) systems.

The quenching constant, *K_SV_*, is determined by the Stern-Volmer Equation (1) [18]:

(1) $$\frac{F_{0}}{F}=1+K_{SV}[Q]$$

where *F*_0_ and *F* are fluorescence intensities of anti-hFABP in absence and presence of the quencher (HNBID or FITC), respectively. Stern-Volmer plots on the linear domain are displayed in Figure 3a and the resulting *K_SV_* values are given in Table 1. One can observe that HNBID has an approximately 2.5 higher protein quenching ability when compared to FITC. This translates into lower amounts of HNBID being needed to obtain the same effect as FITC, and thus into a smaller perturbation exerted upon the studied system.

The binding parameters are determined employing the Scatchard equation in non-linear form (2), considering the presence of either one (*i* = 1) or two (*i* = 2) independent sets of equivalent binding sites [19,20]:

(2) $$\nu =\sum_{i}\frac{n_{i}K_{i}{\left[ligand\right]}_{free}}{1+K_{i}{\left[ligand\right]}_{free}}$$

where *ν* = [ligand]_bound_/[anti-hFABP] is the mean degree of binding, *i.e.*, the number of moles of ligand bound per mole of protein. The concentration of free ligand is estimated by Equation 3:

(3) $${\left[ligand\right]}_{free}=[ligand](1-\frac{F-F_{\infty }}{F_{0}-F_{\infty }})$$

where [ligand] is the total HNBID or FITC concentration, *F_0_* and *F* are fluorescence intensities in absence and presence of the ligand, respectively, and *F*_∞_ the fluorescence intensity of anti-hFABP at fully bound ligand. Statistically significant fits were obtained for Equation 2 when *i* = 2, indicating, for both ligands, the presence of two classes of binding sites: the first class consists of one binding site (*n*_1_ = 1), *i.e.*, the high affinity site (h.a.s.), while the second class encompasses several binding sites (*n*_2_ = 4 for HNBID and 10 for FITC) with affinities one order of magnitude lower than that of the h.a.s. (Table 1). Additional confirmation of the presence of more than one class of binding site has been obtained by observing a non-linear representation (Figure S1) when plotting the Scatchard equation in linear form. By means of molecular modeling, we attempted to localize the h.a.s., as well as some of the lower affinity binding sites (Section 2.3.).

#### 2.1.2. Synchronous Fluorescence

**Figure 4:**
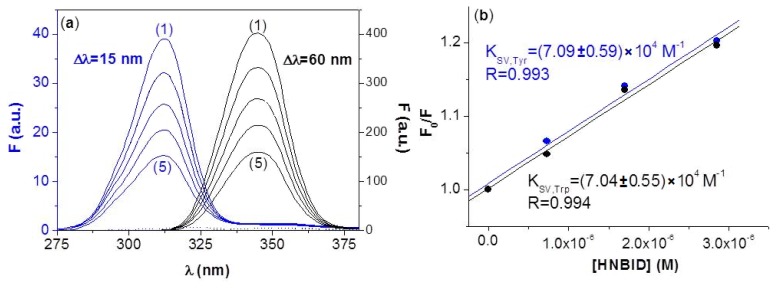
(**a**) Synchronous fluorescence spectra of anti-hFABP (0.85 μM) in absence (1) and presence (2–5) of increasing amounts of HNBID (in the range 0–11 μM). Dotted spectra: [HNBID] = 30 μM; (**b**) Stern-Volmer plots.

The synchronous fluorescence spectra of the Tyr and Trp residues in anti-hFABP, in presence of HNBID, are displayed in Figure 4a. Similar spectra in presence of FITC have been recorded and are given in Figure S2a in the Supplementary Material. Differently from steady-state fluorescence, which offers an overall indication on the quencher’s effect on the fluorescent amino acids, the synchronous technique allows us to estimate to which extent each type of amino acid has been perturbed. The similar *K_SV_* values obtained from synchronous data on the Tyr and Trp bands in presence of HNBID are given in Figure 4b and show that these residues are affected by the ligand to the same degree. In the presence of FITC, a somewhat higher quenching of the Tyr residues has been observed (Figure S2b) in the Supplementary Material), and this correlates to the molecular modeling results indicating the existence of several Tyr residues in close proximity (within 5 Å) of bound FITC (Section 2.3.). Since no synchronous band shifts are observed in the presence of either ligand, one can conclude that neither HNBID, nor FITC cause a major alteration in the polarity around the Tyr and Trp residues.

#### 2.1.3. Time-Resolved Fluorescence

**Figure 5:**
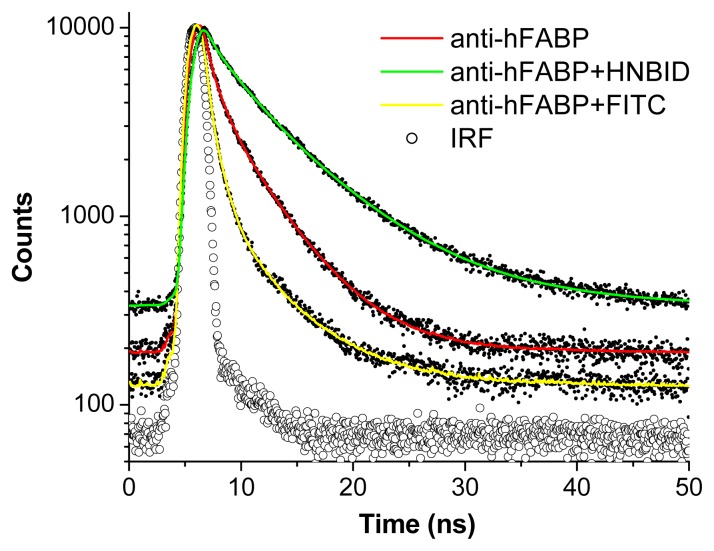
Fitted fluorescence intensity decays of anti-hFABP (0.85 μM) in the absence and presence of HNBID or FITC (11 μM). λ_ex_ = 294.6 nm; λ_em_ = 345 nm; IRF = instrument response function.

The increasing interest on applying fluorescence lifetime measurements to either *in vivo* [21,22] or to biologically relevant *in vitro* samples [23] has prompted us to also investigate the effect of HNBID and FITC on the fluorescence decay characteristics of anti-hFABP. The decays of the protein in absence and presence of ligand (Figure 5) were fitted with triexponential functions (see Experimental section for details) and the computed lifetimes, ascribed to different conformers of the Trp residues [24], are listed in Table 2. It must be stressed that the τ_1_ value is below the resolution of our device and could not accurately be determined. However, on HNBID addition, all three lifetime components of anti-hFABP increase, including τ_1_, which now becomes measurable. This is a feature that could be used to our advantage when performing lifetime measurements on HNBID-labeled antibodies. The effect of HNBID is even more poignant when comparing the values of the mean lifetime, <τ>. It can be observed that the effect of FITC is much smaller, manifested only in an increase in the τ_3_ value.

### 2.2. Effects of HNBID and FITC on the Anti-hFABP Secondary Structure

We aimed to determine to which extent the binding of HNBID and FITC destabilizes the secondary structure of anti-hFABP. The supramolecular structure of IgG1 immunoglobulins is that of a monomeric Y-shaped protein. It consists of four polypeptide chains (two identical heavy chains, B and D, and two identical light chains, A and C) composed of compact, globular domains that are folded into β-sheet and α-helix conformations to different degrees, depending on the immunoglobulin type [25]. Each light chain has two domains (V_L_ and C_L_) and each heavy chain has four domains (V_H_, C_H_1, C_H_2 and C_H_3). Each domain contains two cysteine (Cys) residues that form the intra-domain disulfide bond, and at least one Trp residue [15]. The domains are held together by disulfide bonds and non-covalent forces.

**Figure 6:**
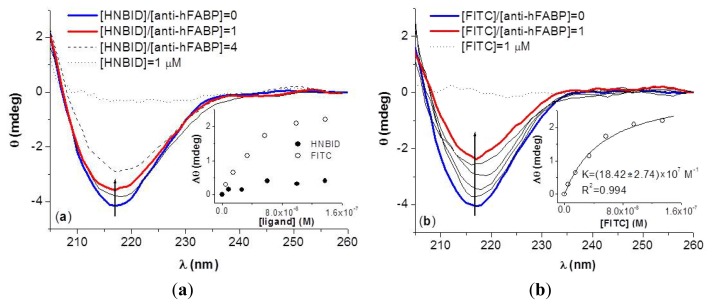
Circular dichroism spectra of anti-hFABP (0.15 μM) in presence of HNBID (**a**) and FITC (**b**). Insets: ellipticity difference, at 216 nm, with respect to isolated anti-hFABP *versus* ligand concentration.

The circular dichroism spectrum of anti-hFABP is that of a typical immunoglobulin, with a negative band at 216 nm revealing a high content of β-sheet structure [26]. Upon ligand addition, the secondary structure of anti-hFABP is altered to a relatively small extent by HNBID, but significantly by FITC (Figure 6). One can observe that, at the same concentration of ligand, there is a two-fold decay in the intensity of the dichroic signal of anti-hFABP in the presence of FITC, while the decrease is only of about 1.2 in the presence of HNBID. These changes in the features of the dichroic spectrum of anti-hFABP in the presence of ligands will be discussed on the basis of molecular modeling results (Section 2.3.).

In the case of FITC, the significant spectral changes observed allowed for the estimation of the binding constant. As the experimental data was collected in the range [FITC]/[anti-hFABP] = 0–1, the non-linear Equation (4) describing the formation of a 1:1 complex [27] was applied for this purpose, and the corresponding fit is given in the inset of Figure 6b. The *K* value obtained (18.42 × 10^−6^ M^−1^) is of the same order of magnitude as that computed from fluorescence data for the h.a.s. of FITC (50.41 × 10^−6^ M^−1^).

(4) $$\Delta \theta =\Delta \theta_{\text{max}}\frac{K[FITC]}{1+K[FITC]}$$

**Figure 7:**
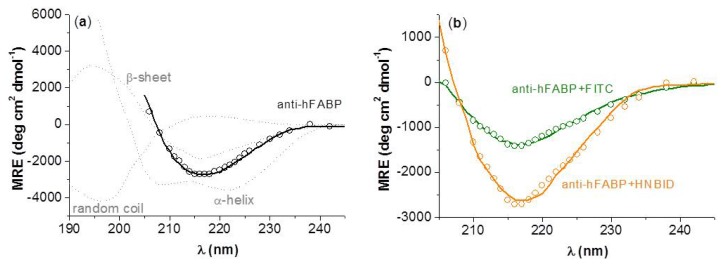
The experimental (line) and simulated (circle) dichroic spectra of anti-hFABP in absence (**a**) and presence (**b**) of HNBID or FITC. Standard β-sheet, α-helix, and random coil poly-l-lysine spectra (the MRE values have been divided by 10) are also depicted for emphasis (**a**, in grey). [anti-hFABP] = [HNBID] = [FITC] = 0.15 μM.

On the basis of the experimental dichroic spectra, the percents of β-sheet, α-helix, and random coil conformations for anti-hFABP in the absence and presence of HNBID and FITC were computed by multiple linear regression analysis, as described in the Experimental Section. The results are given in Table 3, and Figure 7 shows the good agreement between the thus simulated spectra and the experimental ones. One can see that HNBID affects mostly the β-sheet structure of anti-hFABP, while FITC decreases both the β-sheet and α-helix contents. These findings correlate to the molecular modeling results, as will be shown below.

### 2.3. Localization of the HNBID and FITC Binding Sites on IgG1 Anti-hFABP

As stated above, the immunoglobulin structure is Y-shaped, the two arms representing the Fab region. The antigen-binding sites are located within the variable portion (Fv) of each Fab arm, in a small (15–22 amino acids) region at the amino terminal end [28]. We aimed to determine whether the binding of HNBID or FITC occurs in this region and thus affects the ability of anti-hFABP to bind hFABP. The leg of the Y-shaped immunoglobulin represents the Fc region, which contains only constant domains. Since the two Fab arms are identical, only one has been used in our computations (containing A and B chains), alongside the Fc region. The procedure applied for obtaining the lowest energy clusters is described in the Experimental section.

**Figure 8:**
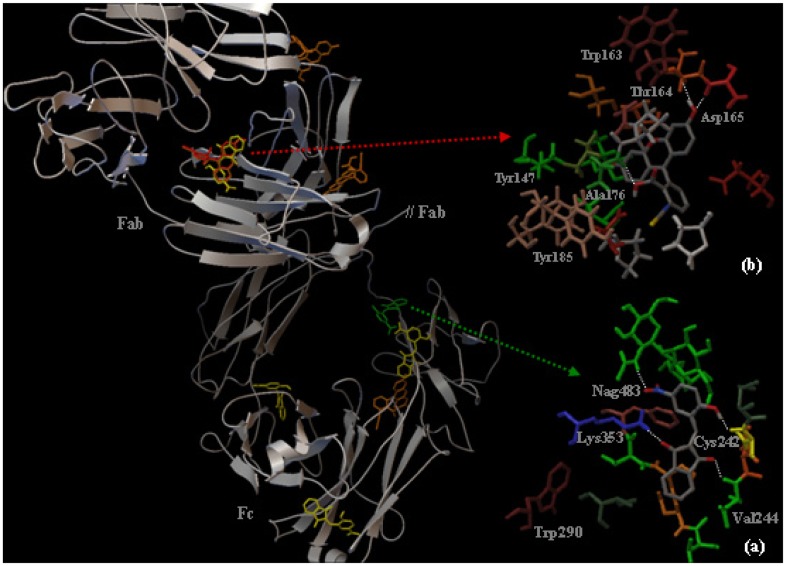
***Left***: The localization in the Fab and Fc regions of IgG1 of the binding sites of HNBID (high affinity binding site in Fc, in green, and low affinity binding sites in Fab and Fc, in yellow) and FITC (high affinity binding site in Fab, in red, and low affinity binding sites in Fab and Fc, in orange). ***Right***: IgG1 amino acid residues within a 5 Å distance from the ligand, for HNBID (**a**) and FITC (**b**); hydrogen bonds are represented by white dotted lines.

In what concerns the lowest energy conformer of the ligand–protein system (localization of ligands depicted in Figure 8 in green for HNBID and in red for FITC), corresponding to the h.a.s., we determined by fluorescence. Our results show that HNBID and FITC bind to different regions of IgG1. HNBID binds to the Fc region, in a pocket along the B heavy chain in close proximity of the glycosidic residues (MAN482, BMA478 and NAG483). This is one of the known binding sites in the Fc segment, located in proximity of the hinge region of the C_H_2 domain [29]. The amino acid residues within a 5 Å distance from HNBID are Ile241, Cys242, Thr243, Val244, Val250, Ser251, Ser252, Val253, Phe254, Ile351, and Lys353, and are depicted in Figure 8a. The Trp residue closest to HNBID is Trp290 in the B chain (10 Å). This complex is stabilized by the formation of four hydrogen bonds in which HNBID acts as either a hydrogen bond donor with Cys242 (via the hydrogen atom of the –OH group in HNBID) or as a hydrogen bond acceptor with Val244, Lys353 and Nag483 (via the two carbonylic and one nitro oxygen atoms, respectively). This affinity of HNBID, for the antibody region containing oligosaccharide chains, could constitute an advantage as the labeling conditions might be milder.

As opposed to HNBID, FITC does not prefer the proximity of glycosidic residues, but binds to the Fab region of IgG1 and is surrounded within a 5 Å distance by residues from both the A light (Glu41, Trp163, Thr164, Asp165, Phe174) and B heavy (Pro41, Val144, Tyr147, Glu150, Pro151, Val152, Val154, Thr173, Pro175, Ala176, Tyr185, Leu187, Ser189) chains, as displayed in Figure 8b. FITC forms three hydrogen bonds, as either a hydrogen bond donor with Asp165 and Ala176 (via the hydrogen atoms of the phenolic and carboxylic –OH groups, respectively), or as a hydrogen bond acceptor with Thr164 from chain A (via the oxygen atom of the carboxylic –OH group). The localization of FITC on the Fab fragment, where the α-helix structure of IgG1 is mostly present [30], together with the deeper protrusion of FITC into the protein pocket, as compared to the binding site of HNBID, are consistent with our circular dichroism results that indicate a higher perturbation produced by FITC upon the protein structure.

Several other conformers with energies two to four kcal/mole higher than the aforementioned ones have been found, and they most probably correspond to the lower affinity binding sites we determined by fluorescence. The four lowest in energy are depicted in Figure 8 (in yellow for HNBID and in orange for FITC). For HNBID, three of these sites are located in the Fc and one in the Fab region, underlining its propensity for Fc binding. Each of them is stabilized by two hydrogen bonds with IgG1 residues and they are located within a 5Å distance from Trp and Tyr residues (Trp332, Trp448, Tyr147, Tyr419), which explains their detection by fluorescence spectroscopy. For FITC, the majority of the low affinity binding sites are located in the Fab region (in the vicinity of Trp163, Tyr147 and Tyr185 residues). This is in accordance to the known decrease in antigen-binding activity of some FITC-labeled antibodies, due to reactions occurring between FITC and amino groups in the Fab region.

## 3. Experimental Section

2-(2-Hydroxy-5-nitrobenzylidene)-1,3-indanedione (HNBID) was synthesized following the protocol described in [31]. Monoclonal mouse anti-human fatty acid binding protein (anti-hFABP) was purchased from HyTest Ltd (Finland).

UV-Vis absorption spectra were recorded on a V-560 Jasco UV-VIS spectrophotometer. Steady-state and synchronous fluorescence spectra were collected with a FP-6300 Jasco spectrofluorimeter (λ_ex_ = 280 nm for steady-state measurements on the anti-hFABP band; Δλ = λ_em_ − λ_ex_ = 15 nm (Tyr excitation) and 60 nm (Trp excitation) for synchronous measurements). The fluorescence quenching titration method was used. 0.85 μM anti-hFABP solutions in phosphate buffer of pH 7.4 were titrated with 30 μM solutions of either HNBID in DMSO, or FITC in DMSO:phosphate buffer 0.5:9.5 volumetric ratio until saturation occurred. Corrections for the inner filter effect of the ligands at the excitation wavelength of the protein were performed [32], as well as dilution corrections.

Time-resolved fluorescence decays were recorded in a time-correlated single photon counting FLS920 system from Edinburgh Instruments (λ_ex_ = 294.6 nm). Data was fitted with a multi-exponential decay. Intensity-averaged lifetimes were calculated according to Equation 5, as in [33]:

(5) $$<\tau >=\frac{B_{1}\tau_{1}^{2}+B_{2}\tau_{2}^{2}+B_{3}\tau_{3}^{2}}{B_{1}\tau_{1}+B_{2}\tau_{2}+B_{3}\tau_{3}}$$

where *τ*_1_, *τ*_2_, *τ*_3_ are fluorescence lifetimes of the excited species and *B*_1_, *B*_2_ and *B*_3_ are pre-exponential factors.

Circular dichroism spectra of 0.15 μM anti-hFABP solutions were recorded on a Jasco J-815 CD spectropolarimeter, in the absence and presence of ligands in the range [ligand]/[anti-hFABP] = 0–1. The scanned wavelength domain was 200–260 nm and the time constant, scan speed, bandwidth/resolution, and sensitivity of the device were set at 4 s, 100 nm/min, 1 nm and 100 millidegrees, respectively. Each spectrum was signal-averaged three times. The corresponding circular dichroism simulated spectra and the percentages of secondary structures present in the systems were determined by multiple linear regression analysis, considering the experimental spectrum of anti-hFABP as being described by Equation 6:

(6) $$[\theta ]={\text{f}}_{\text{H}}{\left[\theta \right]}^{H}+{\text{f}}_{\text{B}}{\left[\theta \right]}^{B}+{\text{f}}_{\text{R}}{\left[\theta \right]}^{R}$$

where [*θ*] is the experimental spectrum (signal intensity expressed in mean residue ellipticity, MRE), f_H_, f_B_ and f_R_ are molar fractions of α-helix, β-sheet, and random coil, respectively (f_H_ + f_B_ + f_C_ = 1), and [*θ*]*_H_*, [*θ*]*_B_* and [*θ*]*_R_* are experimental spectra of a protein reference (poly-l-lysine) in 100% α-helix, β-sheet, and random coil conformation, respectively. These reference spectra were taken from [34]. The MRE values (in units of deg cm^2^ dmol^−1^) were computed by Equation 7 [35]:

(7) $$MRE=\frac{\theta }{10rl[anti\;h-FABP]}$$

where *θ* is the observed circular dichroism (in millidegrees), *r* = 1312 is the number of amino acid residues in anti-hFABP and *l* is the path length of the cell (1 cm).

All measurements were performed at 25 ± 1 °C.

Geometry optimization of HNBID and FITC was carried out by density functional theory calculations using the B3LYP functional, and the 6-31G basis set, in the frame of the GAMESS package [36]. The solvent (water) effect was introduced by the polarizable continuum model [37]. The localization of the HNBID and FITC binding sites on anti-hFABP was investigated with the Autodock 4.0. program [38]. AutoDockTools was used for preparing the protein structure before docking and for visualizing the resulting clusters [39]. The crystal structure of anti-hFABP was taken from Brookhaven Protein Data Bank [40], entry code 1IGY [41]. We investigated the affinity of HNBID and FITC for binding in the Fab and Fc regions of the immunoglobulin. For this purpose, grids (in Å) of 60 × 60 × 60 dimensions with a spacing of 0.386 Å were positioned in several steps to cover the entire aforementioned regions. Ten conformers were obtained in each case using a Lamarkian genetic algorithm and the lowest energy ones were selected for discussion. A total of 140 conformers were considered for each ligand. Details on the docking method can be found elsewhere [42].

## 4. Conclusions

By rationalizing the information gathered by experimental spectroscopic methods (fluorescence and circular dichroism), and the complementary data obtained from molecular modeling, we were able to gain insight into the interaction of the HNBID compound with the IgG1 isotype of mouse anti-human fatty acid binding protein. The parameters characterizing this interaction, namely the quenching and binding constants, classes of binding sites, and excited state lifetimes have been determined. The localization of the high affinity HNBID binding site within the Fc region of anti-hFABP has been evidenced, and the key amino acid and glycosidic residues interacting with the ligand by means of hydrogen bonds, hydrophobic, electrostatic and/or van der Waals forces have been identified. All these data were compared to those obtained performing a similar systematic study on the interaction of FITC, one of the most used fluorescent probes for antibodies, with anti-hFABP. Our results recommend HNBID as a possible valuable alternative to FITC for use as fluorescent label for IgG1 anti-hFABP antibodies. Our further studies will include the labeling of anti-hFABP with HNBID, by taking advantage of the reactive groups on HNBID (–OH or –NO_2_) able to bind covalently to specific functional groups on the Fc region of the antibody, and studying the antigen–antibody interaction, with the ultimate goal of developing a fluorescent sandwich microarray immunoassay for hFABP detection.

## Figures and Tables

**Table 1:** Quenching and binding parameters of the HNBID–anti-hFABP and FITC–anti-hFABP systems.

Ligand	*K_SV_* × 10^−4^ (M^−1^)	*R*^a^; SD b; *N*^c^	*K* × 10^−6^ (M^−1^)	n	*R*; SD; *N*
HNBID	5.75 ± 0.04	0.999; 0.002; 12	41.35 ± 16.85 5.22 ± 0.60	1.00 4.36	0.997; 0.080; 23
FITC	2.25 ± 0.12	0.986; 0.005; 12	50.41 ± 30.59 0.44 ± 0.04	1.01 10.41	0.998; 0.142; 23

Notes:

a *R* = correlation coefficient;

b SD = standard deviation of the fit;

c *N* = number of experimental points.

**Table 2:** Fluorescence decay fitting parameters and intensity-averaged lifetimes of the HNBID–anti-hFABP and FITC–anti-hFABP systems.

System	τ_1_ (ns)	*B*_1_	τ_2_ (ns)	*B*_2_	τ_3_ (ns)	*B*_3_	<τ> (ns)	χ^2^^a^
anti-hFABP	<0.10 *	– *	1.15	0.02	4.47	0.01	2.91	1.684
HNBID–anti-hFABP	0.37	0.02	2.69	0.01	7.33	0.02	6.14	1.373
FITC–anti-hFABP	<0.10 *	– *	1.00	0.03	5.43	0.01	1.74	1.663

Notes:

* could not be estimated with accuracy;

a χ^2^ = statistical parameter of the decay.

**Table 3:** Secondary structure estimation for anti-hFABP in the absence and presence of HNBID or FITC.

System	% β-sheet	% α-helix	% random coil	R^2^
anti-hFABP	88.67	2.09	9.24	0.987
HNBID–anti-hFABP	86.54	2.10	11.36	0.985
FITC–anti-hFABP	82.58	1.09	16.33	0.991

## Supplementary Files

Supplementary File 1 Supplementary Information (PDF, 110 KB)
