# Supplementary material for: 2-(2-Hydroxy-5-nitrobenzylidene)-1,3-indanedione versus Fluorescein Isothiocyanate in Interaction with Anti-hFABP Immunoglobulin G1: Fluorescence Quenching, Secondary Structure Alteration and Binding Sites Localization
Source: Int J Mol Sci. 2013 Jan 31;14(2):3011–25. doi: 10.3390/ijms14023011 (PMC3588028; doi:10.3390/ijms14023011)
Supplement: Supplementary File 1 — Supplementary Information (PDF, 110 KB) [file ijms-14-03011-s001.pdf]

Supplementary Information

Figure S1. Non-linear scatchard plot indicating the presence of more than one class of binding sites for HNBID and FITC on anti h-FABP.

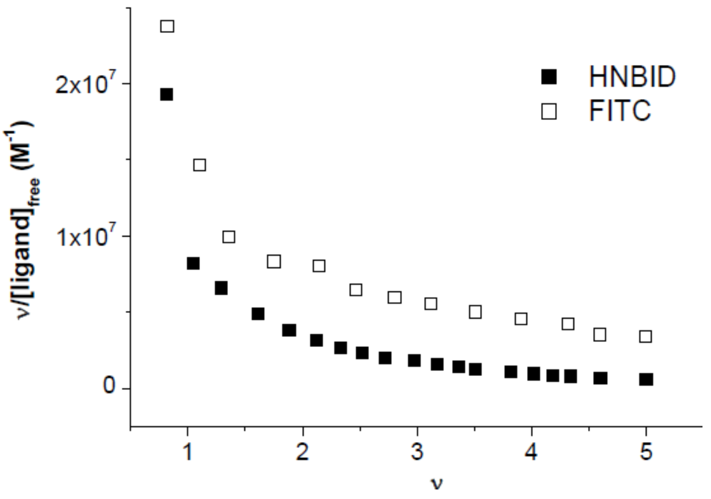

Figure S2. (**a**) Synchronous fluorescence spectra of anti h-FABP (0.85 μM) in absence (1) and presence (2–4) of increasing amounts of FITC (in the range 0–11 μM); (**b**) Stern-Volmer plots.

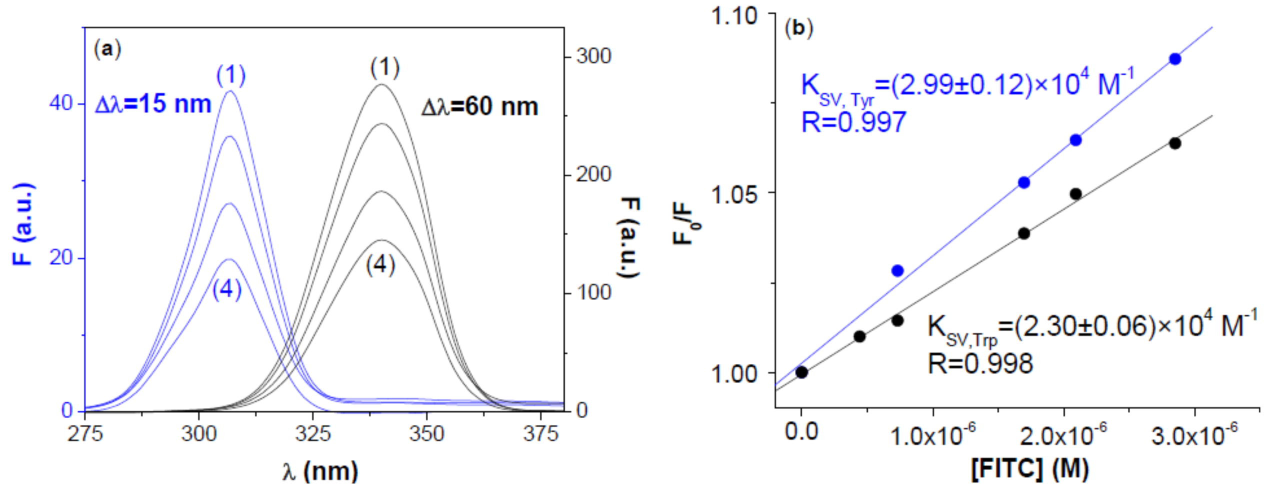

© 2013 by the authors; licensee MDPI, Basel, Switzerland. This article is an open access article distributed under the terms and conditions of the Creative Commons Attribution license (http://creativecommons.org/licenses/by/3.0/).
